# Improving Laser Powder Bed Fusion Printability of Tungsten Powders Using Simulation-Driven Process Optimization Algorithms

**DOI:** 10.3390/ma17081865

**Published:** 2024-04-18

**Authors:** Aurore Leclercq, Vladimir Brailovski

**Affiliations:** Department of Mechanical Engineering, École de Technologie Supérieure, Montreal, QC H3C 1K3, Canada; aurore.leclercq.1@ens.etsmtl.ca

**Keywords:** laser powder bed fusion, tungsten, modeling, numerical predictions, mechanical testing, crack-free specimens, geometric analysis

## Abstract

This study applies numerical and experimental techniques to investigate the effect of process parameters on the density, structure and mechanical properties of pure tungsten specimens fabricated by laser powder bed fusion. A numerical model based on the simplified analysis of a thermal field generated in the powder bed by a moving laser source was used to calculate the melt pool dimensions, predict the density of printed parts and build a cost-effective plan of experiments. Specimens printed using a laser power of 188 W, a scanning speed of 188 mm/s, a hatching space of 80 µm and a layer thickness of 30 µm showed a maximum printed density of 93.2%, an ultimate compression strength of 867 MPa and a maximum strain to failure of ~7.0%, which are in keeping with the standard requirements for tungsten parts obtained using conventional powder metallurgy techniques. Using the optimized printing parameters, selected geometric artifacts were manufactured to characterize the printability limits. A complementary numerical study suggested that decreasing the layer thickness, increasing the laser power, applying hot isostatic pressing and alloying with rhenium are the most promising directions to further improve the physical and mechanical properties of printed tungsten parts.

## 1. Introduction

Tungsten (W) belongs to the group of refractory metals. Among its characteristics are the highest melting point of all metallic materials (3683 K), the lowest coefficient of thermal expansion of all pure metals (~4.6 × 10^−6^ m.m^−1^.K^−1^), excellent mechanical properties at elevated temperatures (tensile strength of ~620 MPa at 1000 °C [1]) and a low evaporation propensity [2]. These unique properties, coupled with radiation impermeability, have significantly increased interest in tungsten, especially in areas of high-temperature applications, such as space, aeronautics and energy (especially nuclear). However, forming tungsten parts by conventional deformation-based technological processes is complicated because of the high ductile–brittle transition temperature range (200–700 °C) and significant sensitivity to oxidation at elevated temperatures (>350 °C).

Due to the above-mentioned difficulties, tungsten parts are mainly shaped in simple geometric forms such as sheets, rods, wires or tubes using powder metallurgy processes such as sintering and hot isostatic pressing [1,2]. To address the challenges that this metal poses in terms of producing parts with complex geometry, new solutions are offered by additive manufacturing (AM) processes, which rely on the use of numerical 3D models to manufacture parts by adding materials in successive layers. Among the AM processes suitable for tungsten parts, laser directed energy deposition (L-DED) and laser and electron beam powder bed fusion (L(EB)-PBF) are the most commonly encountered [3,4]. These three processes are compatible with powder feedstock, which is fortunate since tungsten parts are already manufactured using powder metallurgy, thus making high-quality tungsten powders readily available on the market.

The L-DED process consists of feeding the powdered material through a nozzle into the focus of a laser beam, thus fusing the sprayed powder particles to the substrate, whereas the L(EB)-PBF processes consist of spreading a powder layer on the previously-fused substrate, the new layer being then melted by the laser or electron beam. The L-DED process is mostly used for repairs but has demonstrated very limited success in the production of tungsten parts because of the difficulties involved in fully melting tungsten powders during the deposition. Regarding the two PBF processes, the EB-PBF process offers more powerful heat sources (5–10 kW) and greater build rates than its LPBF competitor (0.2–1 kW) with a lesser need for supports, which enables the stacking of parts during printing. Moreover, this process allows for the reduction in cooling rates and residual stresses, thus limiting the risk of distortion and cracking. On the other hand, the LPBF process provides a higher resolution and better surface finish, and LPBF printers are less expensive for buying and operating than their EB-PBF competitors [5,6]. As examples of the current use of LPBF printers to manufacture tungsten parts, one can mention collimators, radiation shields (Wolfmet 3D, M&I Materials Ltd., (Manchester, UK) [7]) and anti-scatter grids (M 100, EOS GmbH (Munich, Germany) [8]).

In more detail, LPBF relies on fusion by a laser of a 20–60 µm thick layer of metallic powder deposited on a baseplate that can sometimes be preheated to up to 800 °C. Once the layer is melted, the baseplate descends with a controlled increment (one layer thickness), and the operation is then repeated until the final part is obtained. To limit the risk of oxidation, this process takes place under a pressurized and inert atmosphere (often argon, with a typical residual oxygen content of around 800 ppmv) [9,10]. Although promising for the shaping of tungsten, LPBF also presents its share of challenges and limitations. Actually, porosity (voids ranging from 5 to 500 μm in size), cracks and oxidation at the grain boundaries or inside the pores are among the most frequent defects induced during the process [3,4]. To date, pure tungsten parts have been produced with the highest claimed density of 99.6% but generally contain cracks and voids [11]. Despite the presence of process-induced defects, the mechanical properties of printed tungsten parts could be comparable or even competitive to those from conventional forming processes [12]. The difficulties of forming this metal by LPBF are attributable to the multitude of process parameters involved: more than a hundred of them directly or indirectly influence the quality of parts produced, and they can be grouped by category, depending on whether they are related to scanning strategy or to the laser, powder bed or printing chamber characteristics.

Numerous studies have been conducted to understand the effects of process parameters on the properties of printed parts and thus to optimize the process and facilitate the implementation of new printable materials. To reach these objectives, different experiment- and computer-based approaches have mainly been used, the first leading to phenomenological models of the process and the second to numerical or analytical models of the physical phenomena involved. The design of experiment (DOE) approach is the most frequently used to optimize the LPBF process. For example, in [13], the DOE approach was initially used to assess the effects of powder morphology on the characteristics of pure tungsten parts. The authors compared the surface morphology and absorptivity of molten tracks formed by polyhedral and spherical powders and concluded that spherical powders having higher laser absorptivity than their polyhedral counterparts allowed for the formation of continuous tracks. Next, they used the same experimental approach to optimize the LPBF printing conditions (laser power and exposure time) for spherical powders and succeeded to print 96% density parts (measured according to Archimedes’ principle).

Note that for the sake of process optimization, LPBF printing parameters are frequently compounded via the use of a single agglomerated metric, such as the volumetric energy density:

(1) $$VED\;[J.mm^{-3}]=\frac{P}{vht}$$

where P [W] is the laser power, v [mm.s^−1^] is the scanning speed, t [mm] is the layer thickness and h [mm] is the hatching space. Some authors [12,14] studied the impact of this metric on the density and mechanical properties of printed parts by successively varying the laser power and scanning speed while maintaining the layer thickness and hatching space fixed, while others [11,15] focused on varying the scanning speed and hatching space by maintaining the laser power and layer thickness fixed. The problem in using VED as a single process-related metric is that for the same value of VED, the outcomes may differ significantly [16,17]. This notwithstanding, the advantages of experimental methods lie in their relative simplicity and in the possibility to carry out in situ or post-mortem control of printed parts. Because of these benefits, experimental approaches continue to be extensively used and can now benefit from the modern trends in experimental techniques, such as the use of deep learning algorithms that easily convert large amounts of data into phenomenological models also known as numerical twins [18,19]. However, these approaches are costly in terms of materials and time, and the results obtained are difficult to transfer from one piece of equipment to another and from one feedstock material to another.

To decrease the resources needed to implement a new material in the LPBF portfolio, different physical phenomena involved in the LPBF process, such as powder spreading or interactions between the laser and the powder bed, can be simulated using finite element-based models of different complexity [20,21,22,23,24], or analytical models based on exact resolutions of known mathematical equations [25,26,27,28,29]. For example, a finite element-based model was used in [21] to simulate laser–powder bed interactions for pure tungsten powders. This model allowed for the printing of high-density tungsten parts, but these parts contained multiple cracks caused by thermal stresses. This cracking phenomenon was investigated in [22,24] using different FEM approaches and in [26] using a more complex thermo-mechanical coupled dislocation-based crystal plasticity simulations. Despite the models’ complexity and a deep understanding of the different phenomena involved, none of these works allowed for the production of crack-free tungsten parts. On the other hand, simplified analytical models of the process were used in [27,29] to optimize the LPBF process parameters for various metallic materials, such as pure molybdenum, nickel, titanium, iron and aluminum alloys, but, to the best of the authors’ knowledge, the potential of these models has never been explored for LPBF of tungsten parts.

This study was devised to bridge this knowledge gap while trying to propose a cost-effective way of producing LPBF tungsten parts with good structural and mechanical properties. To reach this objective, analytical melt pool simulations and DOE are combined to study the effect of process parameters on the physical, structural and mechanical properties of printed specimens and to suggest some future directions for process improvement.

## 2. Materials and Methods

**Figure 1:**
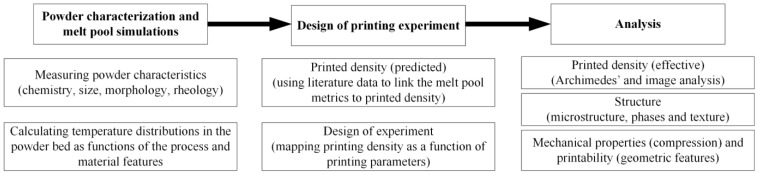
Summary diagram of the methodology followed in this study.

The following workflow was established for this study (Figure 1): first, a selected powder feedstock was characterized in terms of powder granulometry, morphology, flowability and physical properties. Then, an analytical model of the melt pool formed in the powder bed by a moving laser beam was used to define the plan of printing experiments. Next, printed specimens were characterized in terms of their density, structure and mechanical properties. Results of the process simulations were compared with their experimental equivalents, and the LPBF processing parameters were linked to the physical and mechanical properties of the printed specimens. Finally, using the optimized set of printing parameters, selected design artifacts were printed and analyzed to characterize the printability limits.

### 2.1. Powder Characterization

**Figure 2:**
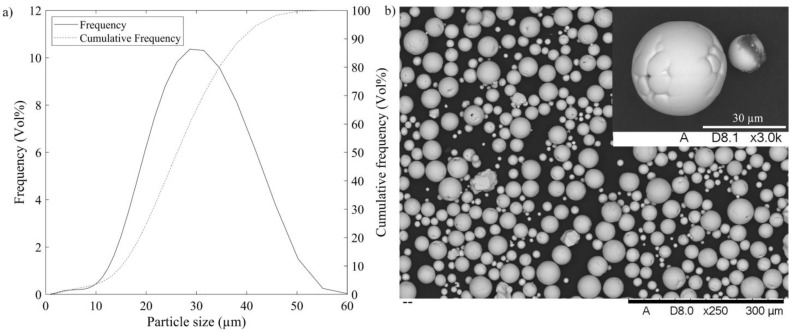
Geometric characteristics of the tungsten powder: (**a**) particle size distribution and (**b**) particle morphology.

Plasma-spheroidized pure tungsten powders (W > 99.9%, O < 250 ppm, tap density > 10 g.cm^−3^ and Hall flow < 8 s/50 g) used in this work were produced by Tekna Co., Ltd. (Sherbrooke, QC, Canada). The particle size distribution (µm) was measured using a Beckman Coulter (Brea, CA, USA) LS13 320 XR particle size analyzer (D_10_ = 16, D_50_ = 27 and D_90_ = 41) (Figure 2a), and the particle morphology was observed using a Hitachi (Tokyo, Japan) 3600 scanning electron microscope (Figure 2b). The rheological properties of dry powders were obtained using an FT4 powder rheometer (Freeman Technology, Tewkesbury, UK). The results are shown in Figure 2 and collected in Table 1.

### 2.2. Building the Printed Density Processing Map

#### 2.2.1. Theoretical Considerations

The temperature field created by a moving Gaussian heat source in a semi-infinite solid [31] can be expressed using Equations (2)–(4):

(2) $$T\left(x,y,z\right)=T_{0}+\frac{AP}{kr_{f}\pi^{\frac{3}{2}}}\int_{+\infty }^{0}\frac{1}{1+\tau^{2}}exp\left(C\right)d\tau$$

with

(3) $$\begin{array}{c} C=-\left(\frac{\tau^{2}}{1+\tau^{2}}\right)\left({\left(\xi -\frac{Pe}{2\tau^{2}}\right)}^{2}+\eta^{2}\right)-\tau^{2}\zeta^{2} \end{array}$$

(4) $$\begin{array}{c} \begin{array}{c} \xi =\sqrt{2}\frac{x}{r_{f}}\;;\eta =\sqrt{2}\frac{y}{r_{f}}\;\;;\zeta =\sqrt{2}\frac{z}{r_{f}}\;;\mathrm{Pe}=\frac{r_{f}v}{2\sqrt{2}\alpha }\;;\tau =\frac{r_{f}}{2\sqrt{2\alpha dt}}\;;\alpha =\frac{k}{\rho c_{p}}\; \end{array} \end{array}$$

where $T_{0}$ is the baseplate temperature (K); A, the absorptivity; P, the laser power (W); k, the thermal conductivity (W.m^−1^.K^−1^); $r_{f}$, the laser beam radius (m); Pe, the Peclet number; v, the scanning speed (m.s^−1^); α, the thermal diffusivity (m^2^.s^−1^); ρ, the density (kg.m^−3^); $c_{p}$, the mass heat capacity (J.kg^−1^.K^−1^); and dt, the time (s).

This equation involves four material-specific parameters: density, absorptivity, thermal conductivity and specific heat capacity. To keep the model simple, these material-related properties are considered constant throughout the melting process. Since the material used in the LPBF process is not in its bulk form but rather forms a powder bed, three of the four material properties (specific heat capacity is already mass- and, therefore, density-independent) must be corrected, considering an effective powder bed density and interactions between powder particles.

**Powder bed density**

The fractional porosity ϕ was calculated using the relative powder bed density ($\rho_{powder})$, as in Equation (5):

(5) $${\phi =1-\rho_{powder};\rho }_{powder}=\frac{BD}{\rho_{s}}$$

where ϕ is the fractional porosity, $\rho_{s}$ is the material density (kg.m^−3^) and BD (kg.m^−3^) is the bulk density measured using an FT4 powder rheometer (Table 1).

**Powder bed absorptivity**

The powder bed absorptivity was calculated according to [32], where the powder bed was assumed as a porous surface composed of spherical bodies and holes, all at the same temperature. Considering the bodies as “gray” without transmission of radiation, absorptivity is then equal to emissivity (A = ϵ). Thus, the powder bed absorptivity can be calculated using Equation (6):

(6) $$\epsilon_{powder}=S_{h}\epsilon_{h}+\left(1-S_{h}\right)\epsilon_{s}$$

where $\epsilon_{powder}$ is the powder emissivity; $S_{h}$, the surface fraction occupied by pores; $\epsilon_{h}$, the pores emissivity; and $\epsilon_{s}$, the emissivity of a bulk material. The emissivity (absorptivity) of the bulk material $\left(\epsilon_{s}\right)$ can be calculated using the Hagen–Rubens model [31]:

(7) $$\epsilon_{s}=A=0.365{\left(\frac{R_{s}}{\lambda }\right)}^{0.5}$$

where $R_{s}$ is the electrical resistivity of the material (Ω.m) and λ is the laser wavelength (m). This model tends to be more accurate at higher temperatures (close to the melting point) and longer wavelengths (above 1 µm).

Next, $S_{h}$ and $\epsilon_{h}$ can be linked to the fractional porosity of a powder bed and the bulk material emissivity using Equation (8), where these values are considered independent of the size of powder particles. This approach was validated for stainless-steel, titanium, aluminum and cobalt–chromium alloy powders in [33,34].

(8) $$S_{h}=\frac{0.908\phi^{2}}{1.908\phi^{2}-2\phi +1};\epsilon_{h}=\frac{\epsilon_{s}\left(2+3.082{\left(\frac{1-\phi }{\phi }\right)}^{2}\right)}{\epsilon_{s}\left(1+3.082{\left(\frac{1-\phi }{\phi }\right)}^{2}\right)+1}$$

**Powder bed thermal conductivity**

Thermal conductivity calculations assume that the powder bed represents a system composed of spherical bodies and holes [35]. The probability of two particles occurring face to face in two successive sections is considered using Equation (9):

(9) $$k_{powder}=k_{s}{\left(1-\phi \right)}^{\frac{4}{3}}$$

where $k_{powder}$ and $k_{s}$ are the thermal conductivities (W.m^−1^.K^−1^) of a powder bed and a bulk material, respectively. Here again, the relation is independent of the particle size; it is dependent exclusively on the fractional porosity of the powder, and was validated for copper powders of different particle sizes and fractional porosities in [35].

**Simplifications related to the temperature dependence of tungsten properties**

**Figure 3:**
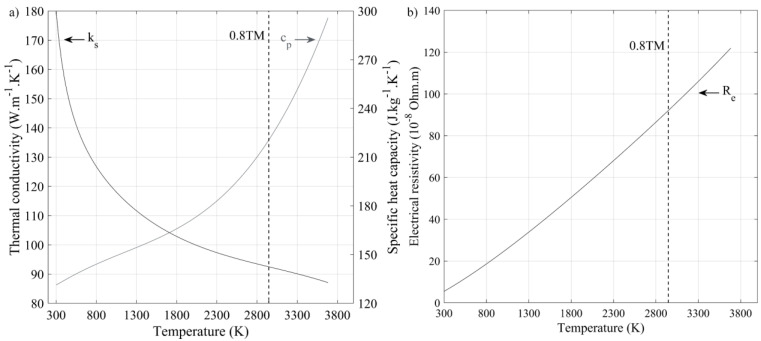
Temperature evolutions of the pure tungsten properties adapted from [36]: (**a**) specific heat capacity and thermal conductivity; (**b**) electrical resistivity.

To simplify calculations, the material properties in the analytical model in Equations (2)–(4) are deliberately considered temperature independent, in spite their effective variations in the room temperature (RT) to melting temperature (TM) range (Figure 3). To implement such an approach, a series of melt pool simulations was carried out by varying the material properties from their RT to TM values (Table 2) and comparing the calculated melt pool dimensions with their experimental equivalents with the objective of selecting material properties that would result in the smallest discrepancies between the calculated and measured melt pool values.

**Figure 4:**
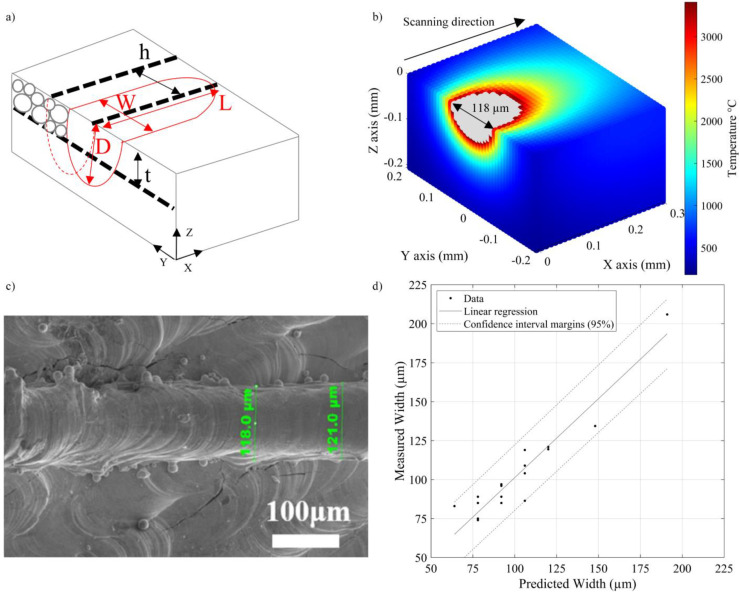
(**a**) Schematic representation of the melt pool, melt pool dimensions (depth D, width W and length L) and process variables (layer thickness t and hatching space h); (**b**) calculated (0.8TM) melt pool for pure tungsten (P = 300 W, v = 400 mm.s^−1^ et r_f_ = 50 µm) and (**c**) photo of a single laser track for the same process parameters from [14]; (**d**) comparison between the measured [11,14,24,37] and calculated (0.8TM) melt pool widths for pure tungsten with 95% confidence margins.

To carry out this analysis, the volume surrounding the melt pool was modeled as a parallelepiped domain (dimensions are in mm): X = [−0.1; 1], Y = [−0.45; +0.45] and Z = [−0.3; 0], with N = 100 divisions for each axis) in the MATLAB software (R2022b, MathWorks, Natick, MA, USA) environment. Next, temperatures calculated in each cell of this domain using Equations (2)–(9) were compared to the melting temperature to extract the melt pool dimensions, and the calculated melt pool values (Figure 4a) were compared to the experimentally measured laser track equivalents. It was found that the smallest discrepancies between the calculated and measured values corresponded to the material properties taken at 0.8TM As an example, the calculated melt pool width for a specific case of P = 300 W, v = 400 mm.s^−1^ and r_f_ = 50 µm with material properties taken at 0.8TM is presented in Figure 4b and compared to its measured laser track equivalent in Figure 4c from [14]. Note also that the calculated melt pool depths were also compared to their experimentally measured equivalents, whenever provided, and showed a decent correspondence. The results of a regression analysis confronting multiple measured laser track widths [11,14,24,37] with their calculated melt pool equivalents (0.8TM) are presented in Figure 4d with a directive coefficient of unity, the negligible constant term, $R_{adj}^{2}=0.90$, RMSE=3 and without any pattern in the residuals. Based on these results, the tungsten properties are selected at 0.8TM. The corresponding powder bed input parameters are therefore as follows: density, 12,100 kg.m^−3^; specific heat capacity, 221 J.kg^−1^∙K^−1^; thermal conductivity, 49 W.m^−1^∙K^−1^; electrical resistivity, 921 nΩ.m; absorptivity, 46%.

#### 2.2.2. Relating the Melt Pool Dimensions to Printed Density

Three dimensionless melt pool metrics (i.e., melt pool depth-to-layer thickness ratio (*D/t*), melt pool width-to-hatching space ratio (*W/h*) and melt pool length-to-width ratio (*L/W*)) were introduced based on the previously described melt pool calculations and process parameters and then related to the relative density of printed parts using experimentally measured density values. The main idea of such an approach can be formulated as follows: to transform a powdered matter into a consolidated body, tracks of molten metal generated by a heat source must overlap in both the horizontal (XY) and vertical (XZ) directions, such as *W/h* and *D/t* ∈ [1.5–2.5]. Additionally, to avoid balling resulting from the melt pool instability, the *L/W* ratio must not exceed 4–4.5 [27].

**Figure 5:**
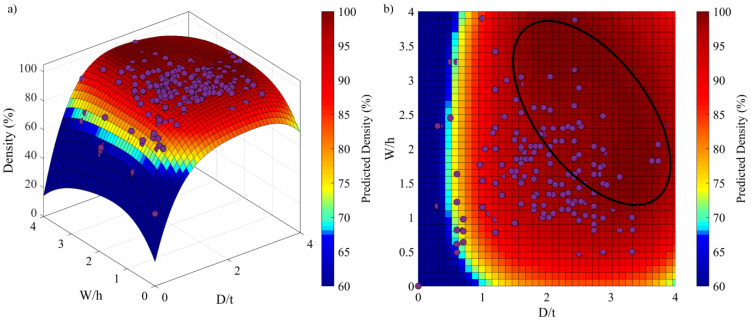
Response surface of the polynomial function relating printed density to dimensionless melt pool metrics (experimental points in purple dots): (**a**) 3-dimensional view and (**b**) *D/t*-*W/h* plane view highlighting a maximum density area defined from the experiment.

To link the dimensionless melt pool metrics (*D/t*, *W/h* and *L/W*) to the printed density, 340 results taken from 19 studies on eight dissimilar materials (W, Mo, In625, Ti64, Ti2225, Fe, AlSi10Mg, AISI 1008) processed using 14 different printers [10,11,12,14,16,17,24,27,33,38,39,40,41,42,43,44,45,46,47] were used to establish a calibration function. As a result of this exercise, a polynomial function presented by Equation (10) relates the calculated adimensional melt pool metrics with the experimentally measured printed densities (coefficients are provided in Table 3, all of them having a significant impact according to their corresponding *p*-values). This function is valid for the laser powder ranging from 70 to 1000 W, the scanning speed from 10 to 2800 mm.s^−1^ and the baseplate temperature from 20 to 1000 °C. The curve fitting was carried out using the “fit” function in MATLAB, choosing the nonlinear least-squares method and the least absolute residuals (LAR) error option. The $R_{adj}^{2}=0.96$, the RMSE=2.0 and the absence of any pattern in the residuals validated the fitting function [48]. The response surface is shown in Figure 5a and its projection on the *W/h*-*D/t* plan in Figure 5b, with the maximum printed density area encircled. Projections of the maximum densities on the *W/h*-*D/t* plan indicate that this response surface meets the established material consolidation criteria, i.e., that a density higher than 95% is obtained when *W/h* et *D/t* ∈ [1.5–3.5].

(10) $$\begin{array}{cc} \rho_{predicted}= & a_{00}+a_{10}\left(\frac{D}{t}\right)+a_{01}\left(\frac{W}{h}\right)+a_{20}{\left(\frac{D}{t}\right)}^{2}+a_{11}\left(\frac{D}{t}\right)\left(\frac{W}{h}\right)\\ & +a_{02}{\left(\frac{W}{h}\right)}^{2}+a_{30}{\left(\frac{D}{t}\right)}^{3}+a_{12}\left(\frac{D}{t}\right){\left(\frac{W}{h}\right)}^{2}+a_{03}{\left(\frac{W}{h}\right)}^{3}\\ & +a_{40}{\left(\frac{D}{t}\right)}^{4}+a_{31}{\left(\frac{D}{t}\right)}^{3}\left(\frac{W}{h}\right)+a_{22}{\left(\frac{D}{t}\right)}^{2}{\left(\frac{W}{h}\right)}^{2}+a_{04}{\left(\frac{W}{h}\right)}^{4} \end{array}$$

#### 2.2.3. Processing Map in the *VED-BR* Coordinates

**Figure 6:**
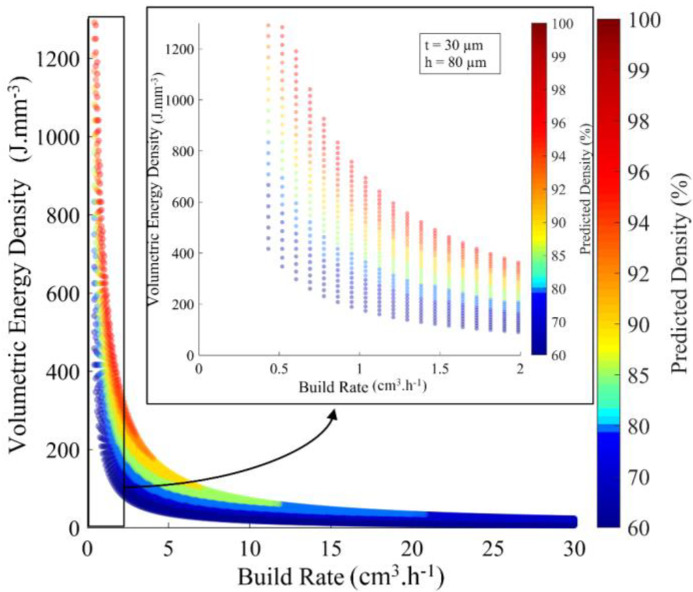
Example of the tungsten density processing map for a TruPrint 1000 (t = 30 µm, h = 80 µm); insert contains the region with the highest predicted density.

Once defined, the analytical model was used to build the “printed density processing map” (Figure 6) in the volumetric energy density (VED, J.mm^−3^) build rate (BR, cm^3^.h^−1^) coordinates where VED was calculated using Equation (1), whereas BR was calculated using Equation (11), and their product corresponded to the maximum power of a laser source (P). Note that this representation allows for avoiding the previously mentioned shortcomings stemming from the use of VED as a single agglomerated metric of the LPBF process.

(11) $$BR\left({\mathrm{cm}}^{3}.h^{-1}\right)=vht$$

Each point on this density map corresponds to a specific set of printing parameters and represents the results of thousands of melt pool calculations carried out within the given process parameter ranges. In fact, Figure 6 shows a process map for pure tungsten corresponding to the capacities of a TruPrint 1000 system (TRUMPF GmbH, (Ditzingen, Germany), $r_{f}=27.5$ µm, λ=1.06 µm), where the laser power P can be varied from 20 to 200 W and the scanning speed v from 20 to 7000 mm.s^−1^. To build this map, the layer thickness t was set at 30 µm, and the hatching space h was set at 80 µm. On this map, the highest densities correspond to *W/h* et *D/t* ∈ [1.5–3.5].

### 2.3. Experiments and Equipment

**Figure 7:**
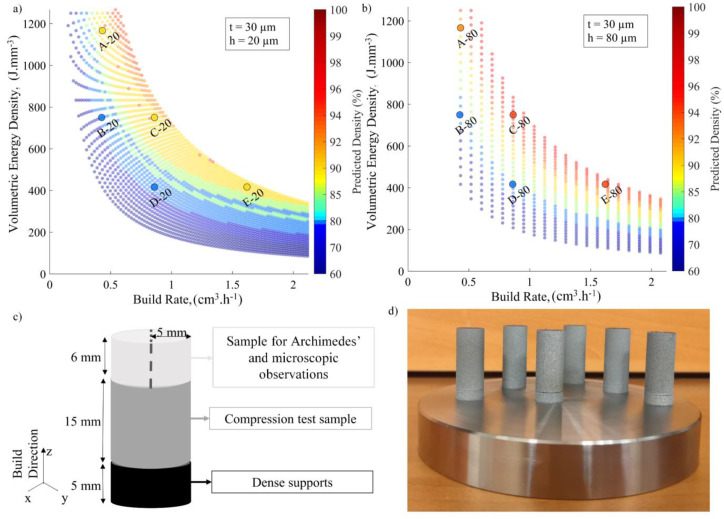
Density maps for tungsten powders with (**a**) h = 20 µm and (**b**) h = 80 µm and selected VED-BR sets for a TruPrint 1000; (**c**) schematic representation of the printed specimens with different cuts; (**d**) photo of a baseplate with printed specimens.

To validate the model predictions and study the impact of process parameters on the physical, structural and mechanical properties of printed specimens, the design of experiments (DOE) approach was applied. The DOE was intended to cover the entire high-printed-density region on the calculated map while being sufficiently large to cross the boundaries of this region by varying the expected printed density from 70 to 95%. To this end, a series of density maps was built for a constant layer thickness of t = 30 µm and a variable hatch distance h (20, 30, 40, 60, 80 and 100 µm), and on each of the maps, five identical VED-BR printing sets, A, B, C, D and E, were defined (Table 4). Examples of such maps are given in Figure 7a,b for the cases of h = 20 µm and h = 80 µm. Each letter corresponds to a given VED-BR set: A, B, C, D, E, and a number corresponds to the hatching space. For example, Specimen A-20 corresponds to a VED-BR set of VED = 1170 J.mm^−3^ and BR = 0.43 cm^3^.h^−1^ and a hatching space of h = 20 µm. The detailed DOE plan of this study can be found in Appendix A, Table A1. On the basis of these assumptions, thirty 10 mm diameter, 20 mm high cylindrical specimens (Figure 7c,d) were printed on a stainless-steel baseplate without preheating. After the printing, specimens were cut from the substrate, supports were removed, and the specimens were cut into different samples for subsequent analysis (Figure 7c).

The density of the LPBF-processed specimens was measured using the Archimedes’ oil impregnation technique [49]. To this end, cylindrical specimens were weighed in air (m_air_), then again after the oil impregnation (m_oil_) and then in distilled water (m_oil+water_) using a density kit (Sartorius (Sartorius Lab Instruments GmbH & Co. KG, Goettingen, Germany) VF4601) and an analytical balance (Sartorius SECURA324-1S with 0.1 mg readability). The specimens’ densities were calculated using Equation (12):

(12) $$\rho_{Arch}=\frac{m_{air}}{m_{oil}-m_{oil+water}}\rho_{water}$$

where the water density (ρ_water_) was taken at room temperature (22 °C).

**Figure 8:**
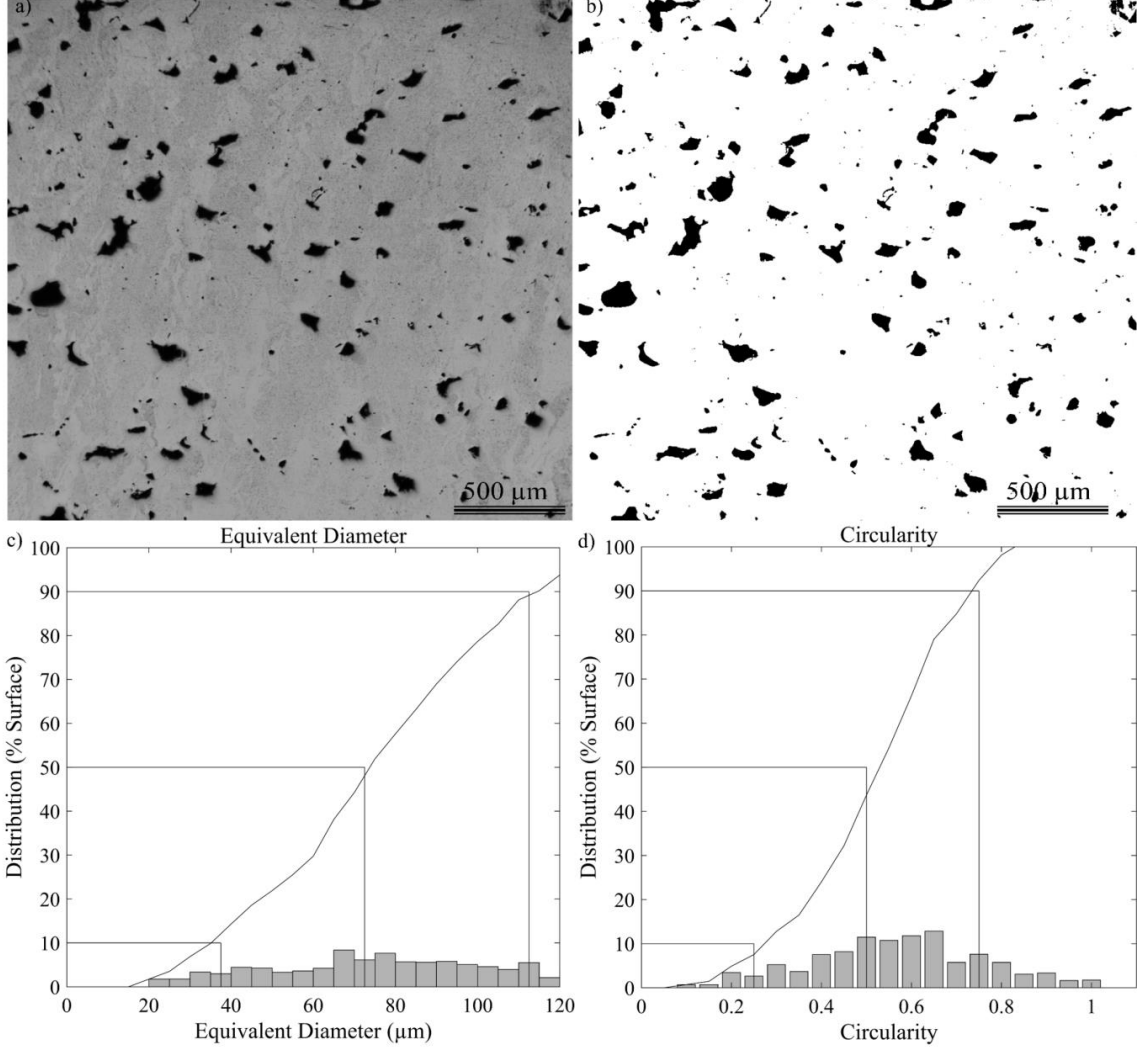
(**a**) LEXT and (**b**) binarized images, (**c**) pore size diagram and (**d**) pore shape diagram for Specimen E-80.

Next, the specimens were mounted in Bakelite, mirror polished and observed with a LEXT OLS4100 (Lext Olympus Corporation, Tokyo, Japan) laser confocal microscope. The density, as well as the number of pores, their sizes and shapes, were extracted using a proprietary routine developed in the MATLAB environment. The following metrics were considered in this study:

- Image analysis material density (ρ_IM_), calculated as a white-to-black pixel ratio in the binarized image; the binarization procedure was applied using the “im2gray” function and a threshold specific to each image. Additionally, the “imfill” function was used to fill the holes and ensure that each pore was entirely black, with no embedded white pixels (Figure 8a,b).
- Pores density (Number of pores per mm^2^, p.mm^−2^), which represents the sum of all the detected pores divided by the image area.
- The 10, 50 and 90% quotients of an entire pore population in terms of their equivalent diameter (DE10p, DE50p and DE90p, µm), obtained by plotting the surface-based distribution of the equivalent pore diameters (Figure 8c). The equivalent diameter of a pore corresponds to the diameter of a circle having the same surface area as the pore, calculated using Equation (13).
- The 10, 50 and 90% quotients of an entire pore population in terms of their circularity (C10p, C50p, C90p, without unit) obtained by plotting the surface-based distribution of the pore circularity (Figure 8d). The circularity of a pore was calculated using Equation (14):

(13) $$Equivalent\;Diameter\;(DE)=\sqrt{\frac{4Area}{\pi }}$$

(14) $$Circularity\;(C)=\frac{4\pi Area}{Perimeter^{2}}$$

In order to focus the study on pores that are the most impactful from the mechanical property viewpoint, those smaller than DE = 20 µm were removed from consideration, which corresponds to removing 75% of the entire pore population in terms of their number, but to only ~5% in terms of their cumulative surface area.

To carry out crystallographic analyses using the electron backscatter diffraction technique (EBSD), specimens were subjected to ion milling using a Hitachi EM 4000 plus (5 kV, 25 rpm, 15 min). The EBSD maps were built using a Hitachi SU-8230 Field Emission-STEM. These maps were then post-treated using the MTEX library (MATLAB software) to assess the grain size, shape, orientation and distribution following the procedure defined in the E2627 ASTM standard [50]. Only the z–y sections along the building direction were analyzed, and the misorientation angle threshold was set to 10°. Since the standard only defines procedures to obtain a number of grains and an average grain size of the entire EBSD map, the grain size and shape metrics were processed by analogy with those of pores and will be referred to as the grain density, i.e., the number of grains per mm^2^ (g.mm^−2^); the grain size DE10_g_, DE50_g_ and DE90_g_ (µm); and the grain circularity, C10_g_, C50_g_ and C90_g_ (without unit). Given that for both the pores and grains, their size and shape distributions are sometimes strongly asymmetrical, their median value (corresponding to 50% of the population) will be considered for future study.

**Figure 9:**
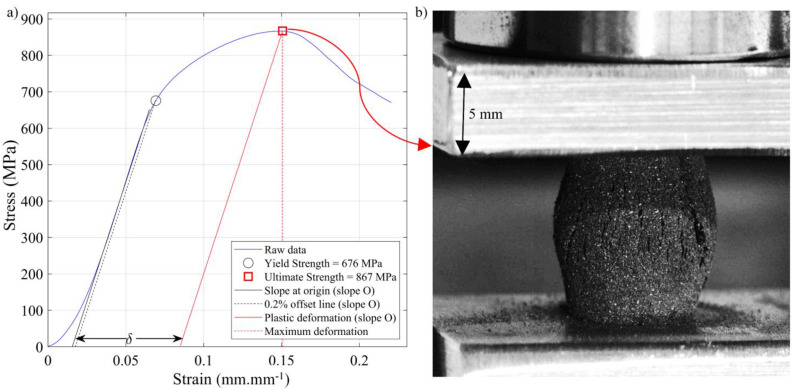
(**a**) Compression stress–strain diagram with the obtained metrics and (**b**) photo of Specimen E-80 during the compression test at a total compression strain of 0.1.

Finally, compression tests were performed using an Alliance RF/200 electromechanical testing machine (MTS, Eden Prairie, MN, USA) according to the E9-09 ASTM standard [51]. The compression rate was set at 0.01 mm.s^−1^, and two high-resolution cameras were placed in front of the specimens during the tests to monitor the fracture mode. The force was measured using an MTS 4501036 load cell, while the displacement was measured by the machine LVDT (since no extensometer was used during testing, the elastic modulus values were not measured). Based on these measurements, strain–stress diagrams were plotted to obtain the following metrics (Figure 9):

- Ultimate compression strength (UCS, MPa), defined as the maximum stress reached during the test.
- Yield strength at 0.2% offset (YS, MPa), calculated by moving the slope at the origin (slope O) to 0.2% on the strain axis, according to the methodology described in the standard.
- Strain under maximum load (δ, %), representing the difference between the total compression strain and the elastic strain to failure, calculated by moving the slope at origin (slope O) to reach the UCS.

Five specimens (A-80, B-80, C-80, D-80 and E-80), all corresponding to h/t = 2.7, were selected for the phase and chemical analyses. First, the XRD analyses were performed using an X’Pert^3^ Panalytical system (Malvern Panalytical Ltd, Malvern, UK) equipped with a cobalt source. Then, in order to detect a possible presence of oxides on fracture surfaces, similarly to [9], the fracture surfaces of 2 of 5 specimens (A-80 and E-80), corresponding to the best and the worst compression properties of this series of specimens, were studied using the XPS X-ray photoelectron spectroscopy technique (Escalab 250Xi (Thermo Fisher Scientific, Waltham, MA, USA) with a monochromatic Al K_α_ source at a power of 218.8 W (14.7 kV, 14.9 mA)) and EDS (Hitachi SU-8230 Field Emission-STEM equipped with an X-flash energy-dispersive X-ray spectroscopy quad detector).

**Figure 10:**
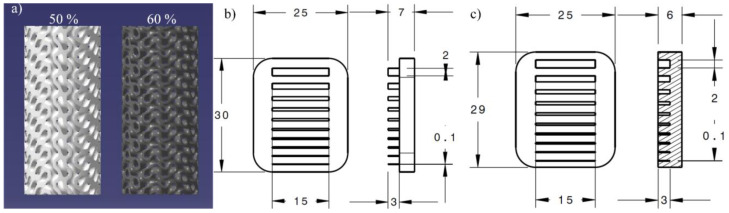
Nominal structure of the printed specimens: (**a**) 50% and 60% porosity gyroid lattice structures, (**b**) minimum wall artifact and (**c**) minimum gap artifact.

Finally, considering the results of mechanical testing, three types of geometric artifacts (Figure 10)—10 mm diameter, 20 mm high gyroid lattice structures with two different porosities (50 and 60%) and the gap and wall printable design features in the 0.1 to 2 mm range (adapted from [52])—were printed using one parameter set leading to the best mechanical properties (E-80). No beam offset or any contour adjustment was made during these prints. The gyroids were characterized in terms of their density through Archimedes’ measurements. No further investigations were conducted on these features because of the high X-ray impermeability of tungsten. The walls and the gaps were measured using a LEXT OLS4100 confocal microscope. All the measurements of the minimum printable structures were repeated 20 times and statistically processed.

In this document, error bars correspond to the confidence interval at 95% for every concerned figure. Sometimes, the error bars are not represented in order to lighten the figure. 

## 3. Results

### 3.1. Archimedes- and Image-Measured Printed Densities

**Figure 11:**
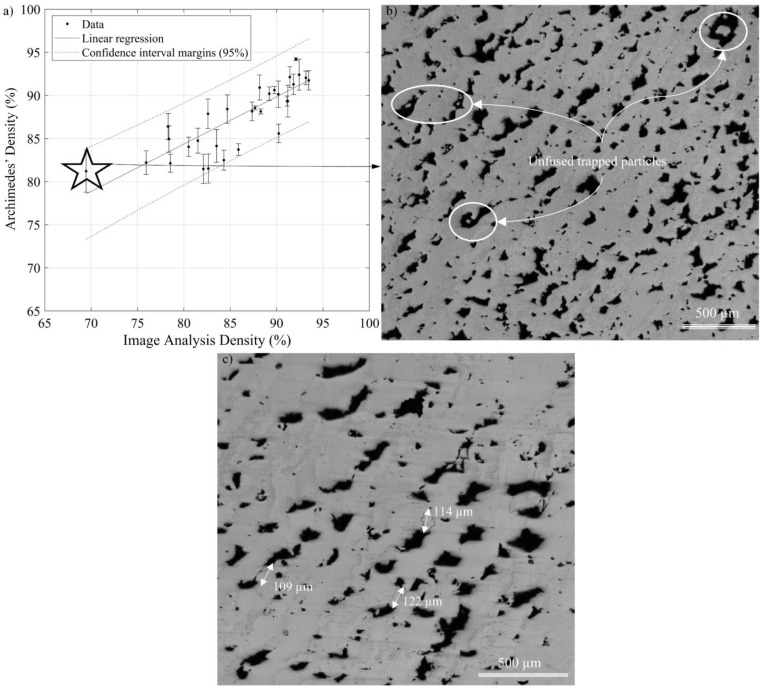
(**a**) Printed densities measured using the Archimedes’ and image analysis techniques, where dotted lines correspond to the 95% confidence interval margins; (**b**) SEM micrograph of Specimen D-20 containing unfused particles inside pores; (**c**) microcracks in Specimen A-40.

In Figure 11a, the results of the Archimedes’ oil impregnation measurements (Archimedes’ density) were compared with those obtained by image analysis (image density). A linear regression analysis between the two groups of results indicates that the difference between them is statistically significant with a confidence interval of 95%. Moreover, the image analysis revealed that pores in the higher-porosity specimens contained unfused powder particles (Figure 11b), thus leading to systematically higher Archimedes’ densities. Since these unfused particles do not contribute to the mechanical resistance of printed parts, relying upon the Archimedes’ measurements may be misleading, and the use of image-based density measurements appears to be more relevant for this study. Furthermore, microscopic observations also highlighted that only two specimens contained microcracks, with an average size of 112 µm ± 3 µm (95%). Since these specimens were printed using h ≤ 60 µm (h/t ≤ 2) and they belong to the A VED*-*BR set with the highest volumetric energy density values and the lowest build rate values, it can be hypothesized that they resulted from high temperature gradients generated during printing. Also, it is possible that these cracks came from the cold work occurring during the metallographic preparation and not from the process parameters, since they were very few and localized (Figure 11c). To validate the second hypothesis, polishing steps were repeated without a definitive conclusion, leaving the hypothesis of excessive temperature gradients as the most probable.

### 3.2. Predicted versus Measured Printed Density

**Figure 12:**
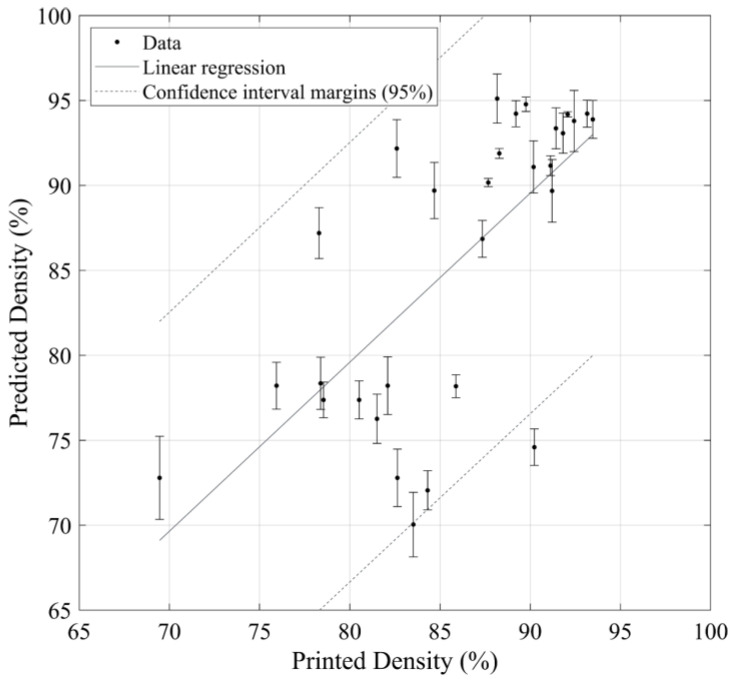
Comparison between the measured and predicted printed densities with the confidence interval margins.

The image-measured and predicted printed densities are compared in Figure 12 and subjected to a linear regression analysis to assess the model relevance, with the following results: the direction coefficient is close to 1 with a *p*-value < 0.05; the constant is not statistically significant according to its *p*-value (*p*-value > 0.05); $R_{adj}^{2}=0.8$; and RMSE=5.2, with no pattern in the residuals. The numerical values of discrepancies between the model and the experiments are presented in Appendix A, Table A1. It is hypothesized that the observed discrepancies are due not only to the model-related limitations but also to the fact that the literature data used to relate the melt pool metrics to the printed density were obtained using the Archimedes’ technique and could be misleading, as previously discussed.

**Figure 13:**
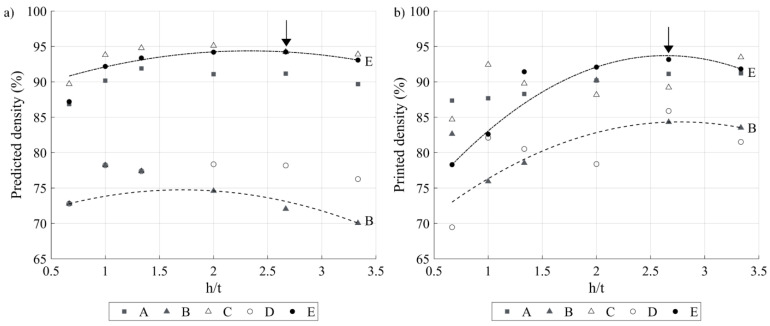
(**a**) Predicted and (**b**) measured printed densities as functions of the h/t ratio for five VED-BR sets (arrows indicate maxima, trend lines are added for visualization purposes).

Next, Figure 13a,b presents, respectively, the predicted and measured printed densities for different VED*-*BR sets as functions of the h/t ratio. It can be seen that for each VED*-*BR set, the predicted and measured densities follow similar trends: they first increase to reach a maximum at h/t = 2.5…2.7 and then decrease. Moreover, for any h/t ratio, the E (417 J.mm^−3^; 1.62 cm^3^.h^−1^) and C (750 J.mm^−3^; 0.86 cm^3^.h^−1^) VED*-*BR sets result in the highest printed densities, while the B (750 J.mm^−3^; 0.43 cm^3^.h^−1^) and D (417 J.mm^−3^; 0.86 cm^3^.h^−1^) VED*-*BR sets result in the lowest printed densities. These results confirm the capacity of the model to relate process parameters and printed densities, both in terms of the trends and absolute values. It can also be asserted, based on these observations, that the processing conditions leading to the highest printed densities, and therefore to potentially higher mechanical properties, correspond to the E-80 (417 J.mm^−3^; 1.62 cm^3^.h^−1^) VED*-*BR set with h/t = 2.7, which gives the following printing parameters: P = 188 W, v = 188 mm.s^−1^, h = 80 µm and t = 30 µm.

### 3.3. Structural Studies

#### 3.3.1. Porosity

**Figure 14:**
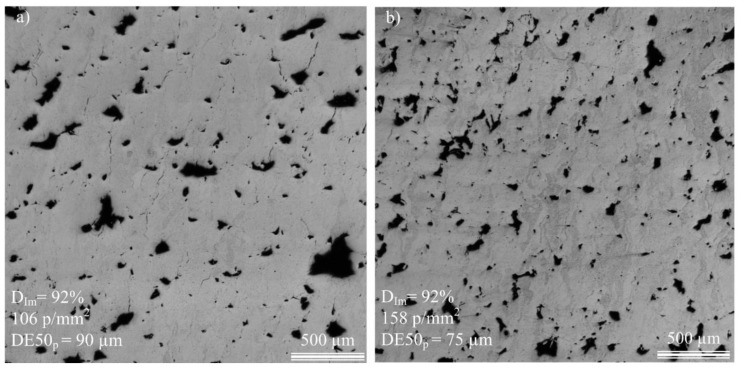
Microscopic observations of (**a**) Specimen E-60 and (**b**) Specimen E-100 having the same printed density of 92% but different pore sizes and pore densities.

**Figure 15:**
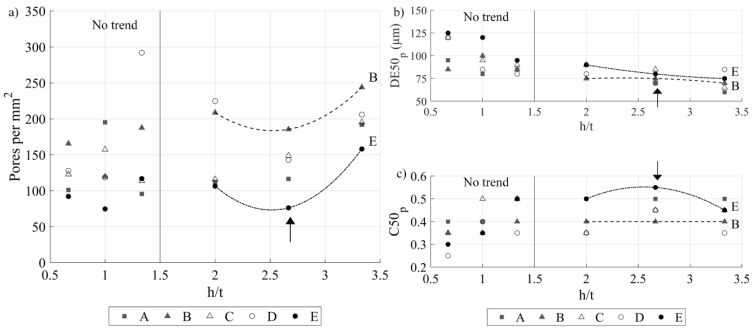
(**a**) Pore density, (**b**) pore size and (**c**) pore circularity as functions of the h/t ratio for five VED-BR sets (arrows indicate extrema, trend lines are added for visualization purposes).

No clear correlation can be established between the pore size and the printed density. For example, as shown in Figure 14a,b, for the same printed density of 92%, Specimen E-60 contains larger pores than Specimen E-100 (90 versus 75 μm), but their number per surface area is fewer (106 versus 158 p.mm^−2^). Some trends, however, become visible when plotting the pore number, size and circularity as functions of the h/t ratio for different VED-BR sets (Figure 15). For h/t > 1.5 (under this value, no trend can be observed), pore number and size follow a trend opposite to that of pore circularity: for each VED*-*BR set, the pore number and size first decrease to reach a minimum at h/t = 2.7 and then increase, while the pore circularity first increases to reach a maximum at h/t = 2.7 and then decreases. As a result, the E set results in the smallest number of pores and their highest circularity, while the B set results in the highest number of pores and their lowest circularity. These two VED*-*BR sets were already established as the best and the worst in terms of the predicted and measured printed densities.

#### 3.3.2. Grain Structure

**Figure 16:**
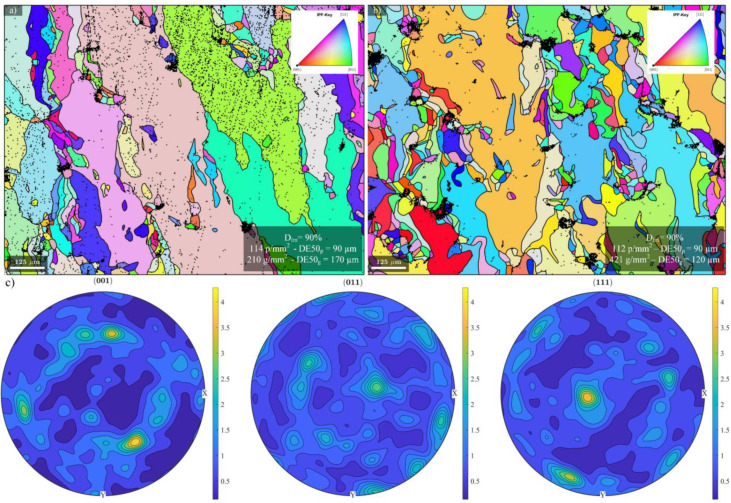
EBSD maps (IPF Y) for specimens (**a**) C-40 and (**b**) A-60 with the same printed density of 92% but different pore and grain structures, and (**c**) example of the pole figures corresponding to Specimen E-80.

Regarding the microstructure, no correlation could be established with either density or number of pores. As illustrated in Figure 16a,b, some specimens with the same printed density manifest different pore and grain structures (D50g ϵ [40; 220] µm). Also, the grains are quite elongated (C50g ϵ [0.2; 0.6]) and tend to follow the build direction. Finally, the crystallographic texture of all the specimens was analyzed, and, as can be seen on the pole figures for Specimen E-80 (Figure 16), no preferential crystallographic orientation was found.

### 3.4. Compression Testing

**Figure 17:**
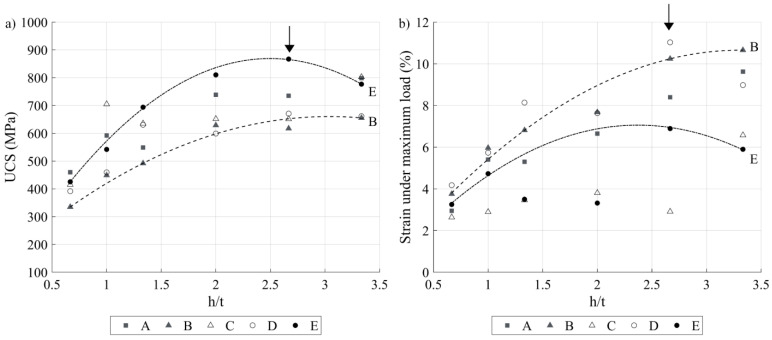
(**a**) UCS and (**b**) compression strain under maximum load as functions of the h/t ratio for five VED-BR sets (arrows indicate maxima, trend lines are added for visualization purposes).

In Figure 17, the UCS values are plotted as functions of the h/t ratio for all five VED*-*BR sets, and it can be observed that, similarly to the printed density (Figure 13b), the UCS first increases to reach the maximum at h/t = 2.7 and then decreases. Also, for any h/t ratio, the E specimens show the highest strength, while the B specimens demonstrate the lowest. Thus, an initial selection of the best process parameters based on the printed density is justified, this combination being VED = 417 J.mm^−3^ and BR = 1.62 cm^3^.h^−1^, with h/t = 2.7 (P = 188 W, v = 188 mm.s^−1^, h = 80 µm and t = 30 µm).

Moreover, even though no quantitative relationships can be established between the compression strength and the pore and grain structures, some worthwhile qualitative observations can be made. Figure 17a shows that the maximum strength is reached at h/t = 2.7 with print set E, when the number of pores is the smallest and their circularity is the highest (see Figure 15a,b). On the other hand, maximum compression strain in Figure 17b was obtained with print set B at the expense of its lower mechanical resistance, which is a logical outcome.

**Figure 18:**
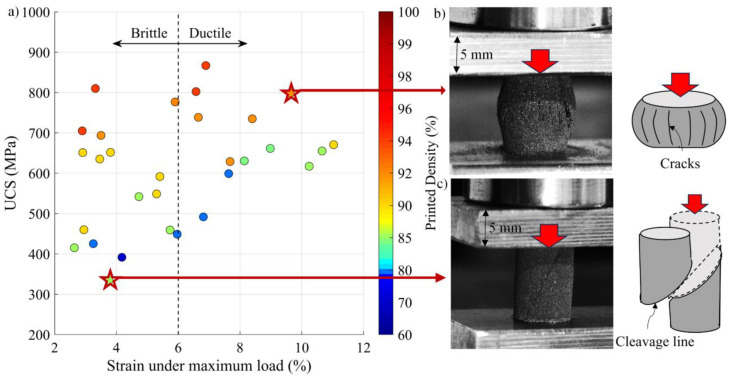
(**a**) UCS versus strain under maximum load (printed density is presented in color) showing a brittle-to-ductile failure separation line at 6%; photo and schematic representation of the (**b**) ductile and (**c**) brittle rupture modes.

In Figure 18, the ultimate compression strength values are plotted as functions of the strain under maximum load. Each dot represents one specimen and is colored according to its measured printed density. Overall, the higher the density, the higher the strength and the lower the strain under maximum load. During compression testing, two rupture modes were observed: in some specimens, a net fracture was observed, oriented at 45° to the compression direction without the crushing typical of the brittle material behavior, and in others, a complete crushing of the specimens was seen with many crack initiations, reflecting a more ductile behavior. These two failure modes could be linked to the measured maximum compression strain as follows: specimens with ductile compression failure manifest a maximum compression strain >6%, whereas specimens with brittle compression failure correspond to a maximum compression strain <6%.

Furthermore, mechanical properties were related to printed densities and the number of pores. A multivariate analysis of variance (MANOVA) showed that a relation between the material strength, printed density and number of pores (Table A2 in the Appendix B) cannot be denied with a 95% confidence interval: the higher the printed density and the smaller the number of pores, the higher the UCS values. However, to decouple the impact on the material resistance of printed density from that of structural features (pore and grain sizes), statistics provided by the present study are not large enough. Finally, in the framework of this study, no relation could be established between the pore and grain structures and the strain at maximum load.

### 3.5. Analyses of the Crystalline Phases and Oxides

**Figure 19:**
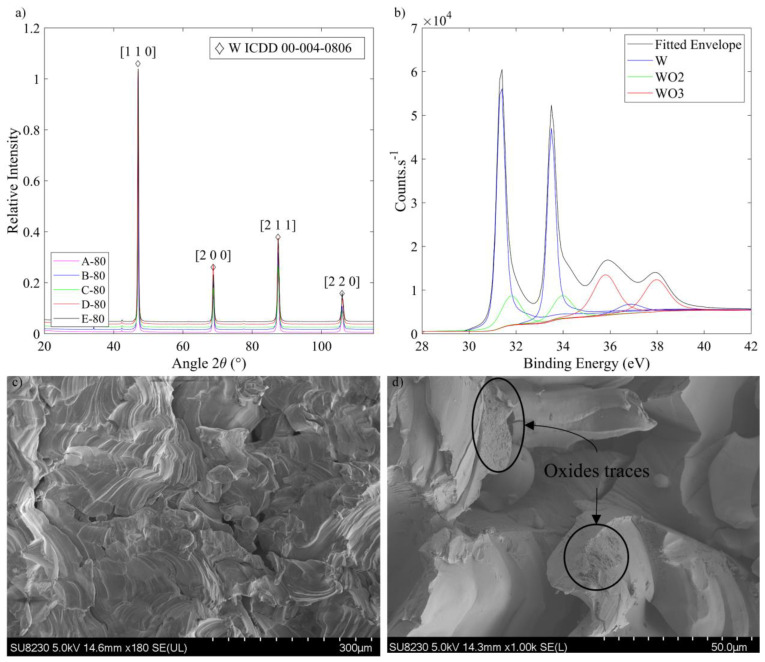
(**a**) XRD analyses of the selected specimens (A-80, B-80, C-80, D-80 and E-80), with four detected crystallographic orientations indicated by a diamond sign; (**b**) XPS analysis of the specimen E-80; (**c**) EDS observation of the fracture surface of Specimen E-80; (**d**) EDS observation with a closer look at some traces of oxides.

It was established that the h/t ratio of 2.7 led to the best physical and mechanical properties overall. Thus, the five corresponding specimens (A-80, B-80, C-80, D-80 and E-80) were selected for the XRD analysis (Figure 19a). The same results were found for all of them: four diffraction peaks could be indexed to the cubic structure of tungsten (ICDD 00-004-0806), but no signs of any oxide could be found. Knowing the limitations of this technique, if there were any oxides, their presence would be less than 5%. To further investigate potential signs of oxidation, the EDS and XPS analyses were conducted on the fracture surfaces of the best (E-80) and the worst (A-80) specimens in terms of their physical and mechanical properties. At first glance, the EDS analysis did not detect any trace of oxygen (Figure 19c). Then, after analyzing the interior of selected regions, some traces of oxygen were revealed (Figure 19d). In these locations, irregularities of the fracture surface were observed, highlighting the potential presence of oxides. This hypothesis was then confirmed by the XPS analyses (Figure 19b), which revealed the presence of WO_3_ (binding energy BE between 35.8–36.1 eV), as well as traces of WO_2_ (BE between 31.8–31.9 eV). Since no quantitative analysis was possible, oxidation can be considered to not have a dominant effect on the causes of rupture of these specimens.

### 3.6. Printable Geometric Features

**Figure 20:**
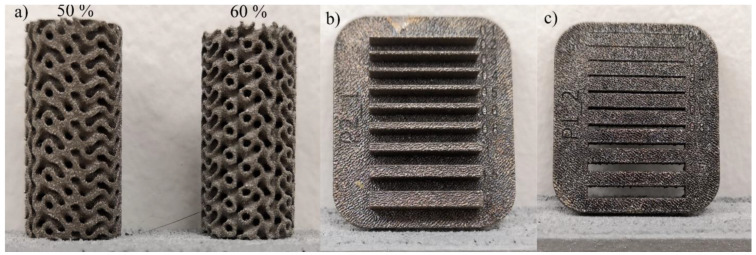
Printed design artifacts: (**a**) gyroids (numbers correspond to nominal printed densities), (**b**) wall and (**c**) gap artifacts.

**Figure 21:**
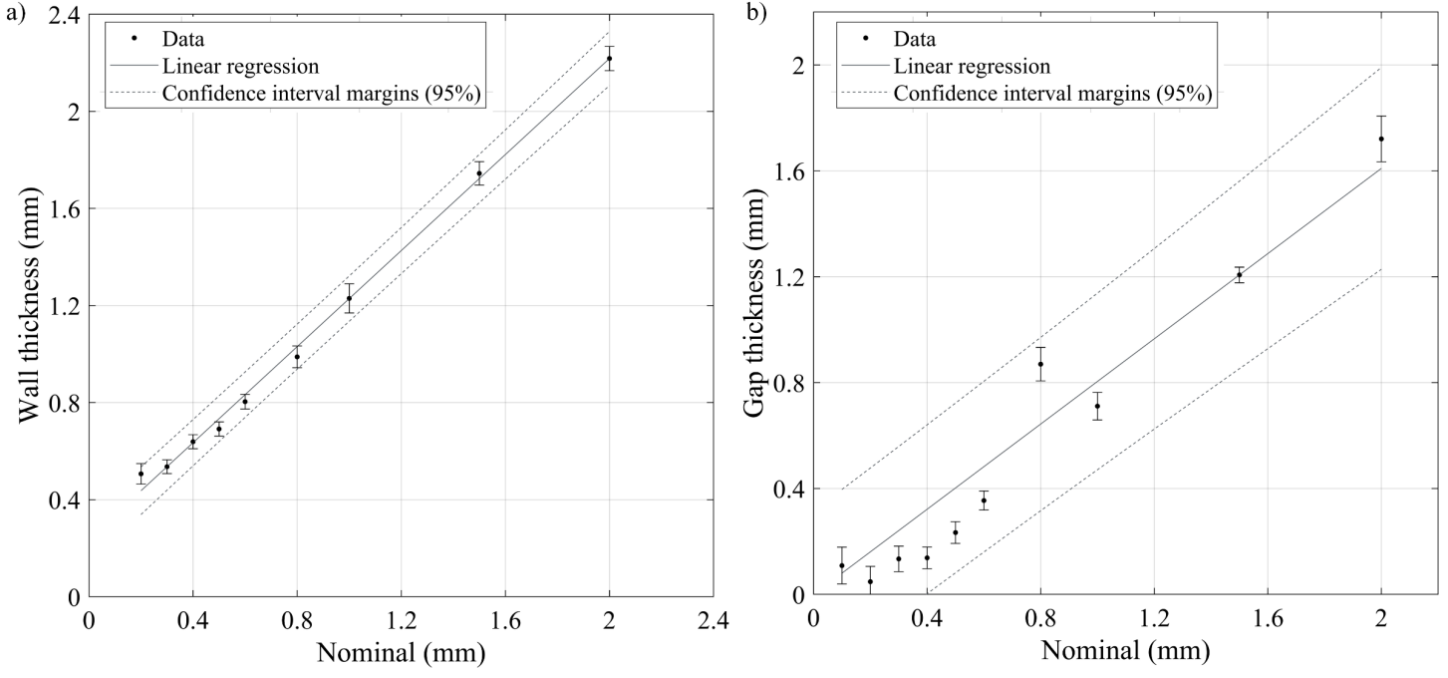
Linear regression between the nominal and the microscope-measured thicknesses of (**a**) walls and (**b**) gaps.

Geometric artifacts presented in Figure 20 were printed with the optimized E-80 parameter set and contain design attributes of interest, i.e., gyroid lattice structures and gap and wall thickness artifacts. The measured relative densities of the lattice structures were 41.2 and 44.2% as compared to the nominal values of 40 and 50%, respectively. Thus, lower porosity gyroid structures manifested a better conformity between the printed and nominal density values. The minimum printable gap corresponded to 0.1 mm, whereas the minimum printed wall corresponded to 0.2 mm. To assess the printing resolution, the measured and nominal dimensions of both the gaps and the walls were compared and subjected to a linear regression analysis (Figure 21). For the walls and the gaps, the direction coefficients were close to 1 (0.99 and 0.89, respectively, with a *p*-value < 0.05), as well as the $R_{adj}^{2}$ (0.99 and 0.94, respectively), and the RMSE values were 0.04 and 0.14, respectively, with no patterns in the residuals. The constant was not statistically significant for the gaps (*p*-value > 0.05) but amounted to 0.24 mm for the walls, meaning that they were ~0.2 mm thicker than their nominal values. A beam offset adjustment must be considered to rectify this systematic error.

## 4. Discussion

**Figure 22:**
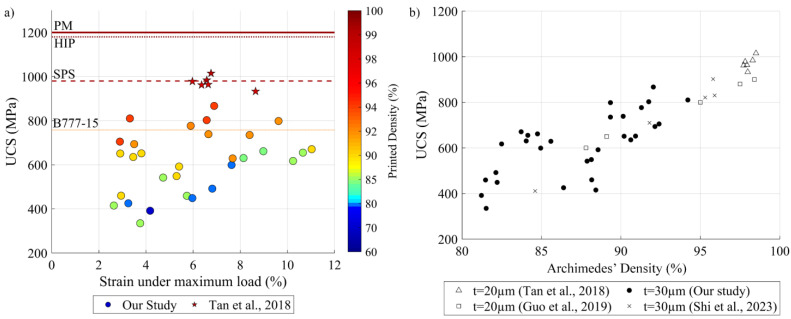
(**a**) Comparison of the mechanical properties of pure tungsten LPBF specimens (our study and [12]) and tungsten parts formed by other processes [12]; (**b**) UCS vs printed density of LPBF tungsten specimens for different layer thicknesses (data from [12,16,44]).

The present study allowed for the maximization of the printed density and mechanical properties of pure tungsten specimens produced from a specific powder feedstock (µm) (D10 = 16, D50 = 27 and D90 = 41) on a given LPBF printer (TruPrint 1000 from Trumpf). To determine the relevance of our results, comparisons were made first with an industrial standard for tungsten parts produced by powder metallurgy B777-15 [53] and then with the literature. This standard for “machinable, high-density tungsten base metal produced by consolidating metal powder mixtures, the composition of which is mainly tungsten” contains requirements for the mechanical properties and chemical composition of generic tungsten-based parts for their use at room temperature. The first comparison reveals that some specimens printed in this study reached classes 1 and 2, which correspond to the most stringent requirements for tungsten parts. However, this comparison must be interpreted with caution since the standard stipulates the tensile resistance of tungsten parts, while in present study, compression properties were measured and no requirements for these properties were found in the literature. Furthermore, the mechanical properties of specimens obtained in this study were compared with the best published data on pure tungsten LPBF parts [12], as well as with the reference data resulting from other tungsten forming processes (Figure 22a). It can be seen that results of the present study are relatively close to those from LPBF and spark plasma sintering (SPS) [12] but sufficiently far apart from those of other processes, such as hot isostatic pressing (HIP) and powder metallurgy (PM).

Figure 22b presents a more detailed comparison between the UCS values of this study and those from the LPBF literature [12,16,44]. It appears that for an equivalent relative density, specimens with thinner layers manifest a higher mechanical resistance. Moreover, in most studies, specimens are printed using more powerful LPBF printers, more specifically 400 W laser printers, versus the 200 W laser TruPrint 1000 unit used in the present study. Two main avenues for improvement of the mechanical properties of printed tungsten parts can therefore be contemplated: (a) through the use of finer powders, and therefore thinner layers, and (b) through the use of more powerful LPBF systems.

To investigate the potential impact of using a more powerful laser, such as 400 W (example M290 of EOS) as compared to 200 W (example TruPrint100 of Trumpf), and a thinner layer, such as 20 μm as compared to 30 μm, two numerical studies were carried out using the model developed in this study.

**Figure 23:**
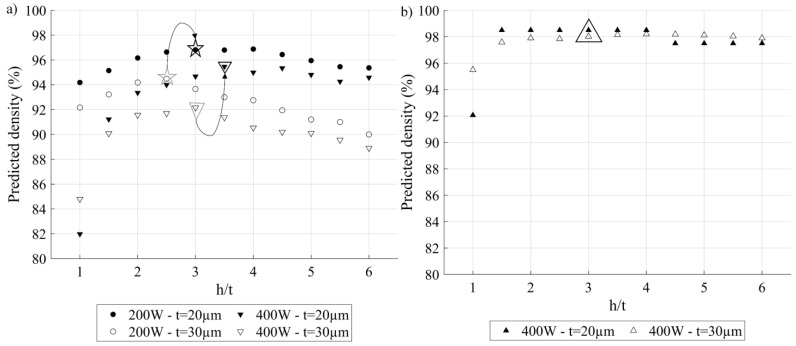
Predicted density for two layer thicknesses (20 and 30 µm) as a function of the h/t ratio: (**a**) using the VED-BR set E (417 J.mm^−3^; 1.62 cm^3^.h^−1^) optimized for the 200 W laser and (**b**) using the VED-BR set (444 J.mm^−3^; 3.24 cm^3^.h^−1^) specifically optimized for the 400 W laser.

For the first study, two different lasers (200 W) and (400 W) and two different layer thicknesses (t = 20 and 30 µm) were considered. The process parameters used for both lasers were the same and corresponded to the E VED*-*BR set identified as having the best processing conditions from the results of previous experiments with a 200 W laser (417 J.mm^−3^; 1.62 cm^3^.h^−1^). Moreover, the powder feedstock properties were also kept unchanged, and that despite the fact that this enabled the use of thinner layers, finer powder feedstocks than that used in this study must be considered. From this first study, the following observations can be made (see Figure 23a):

- For both layer thicknesses and both lasers, the predicted printed density evolves similarly: it increases as a function of the h/t to reach a maximum and then decreases. Globally, the thinner the layer thickness, the larger the recommended h/t ratio, irrespective of the laser power. In more detail, for the 200 W laser, when the layer thickness decreases from 30 to 20 μm, the recommended h/t value increases from 2.5 to 3, (stars, Figure 23a), and for the 400 W laser, when the layer thickness decreases from 30 to 20 μm, the recommended h/t value increases from 3 to 3.5 (triangles, Figure 23a).
- Printing with both devices using the same *VED-BR* set leads to the following conclusion: the thinner the layers, the higher the predicted density, where the maximum predicted density for a 200 W laser is 97%, while for a 400 W laser, this density is 95%.

The last observation is surprising, given the higher available laser power of the 400 W printer. The explanation for this discrepancy is, however, simple: it is the use with the 400 W printer of the printing parameters optimized for the 200 W printer. Knowing that the two systems have different beam spot diameters (55 μm for the 200 W laser and 100 μm for the 400 W laser) and different maximum laser powers, it is not surprising that the optimal parameters for the two systems are different. Thus, a second numerical study has been carried out to estimate a possible increase in the printed density when using the 400 W printer with its specific optimized process parameters (444 J.mm^−3^; 3.24 cm^3^.h^−1^) but different layer thicknesses (30 and 20 μm) (Figure 23b). From this study, the following observations can be made:

- Trends of the predicted density according to the h/t ratio are the same as in the previous numerical study. Also, for the 400 W printer, h/t leading to the maximum predicted density is 3, irrespective of the layer thickness, meaning that no improvements can be made to 400 W printers using thinner layers.
- Considering the optimal printing parameters for each of the printers, the 400 W printer leads to a higher maximum predicted density than the 200 W printer: 99 as compared to 97%.

Based on the preceding, it can be assumed that the density of printed parts, and therefore their mechanical strength, can be increased by reducing the layer thickness and increasing the laser power (to be experimentally verified in a future study). Moreover, reducing layer thickness would also improve the print resolution, as reported in [30]. Using the results of these two studies, the optimized sets of printing parameters leading to the maximum predicted densities potentially reachable on both devices are shown in Table 5.

Reducing the number of structural defects and controlling the microstructures are other ways to improve the physical and mechanical characteristics of printed tungsten parts. According to the Hall–Petch relationship, the smaller the grain size, the higher the mechanical strength [54]. However, since refractory metals are mainly used for high-temperature applications, it is more reasonable to decrease the number of grain boundaries, thus moving towards single crystal-like textures [55,56,57,58,59,60,61,62]. Furthermore, the mean measured grain size range of this study is ~100 µm, which leads to a ductile–brittle transition temperature (DBTT) ranging between 500 and 700 K, which for its part corresponds to the maximum reported DBTT temperature [60,63]. To decrease the DBTT temperature, either ultrafine or single crystal-like grain structures are both legitimate targets. For the same reasons as previously mentioned with respect to the strength, a single crystal-like microstructure should be privileged. Therefore, HIP treatment is potentially well-suited to coarsen the microstructure of LPBF tungsten specimens while simultaneously reducing process-induced porosity. Even if there are only few studies on the impact of HIP on the LPBF tungsten parts, some of them already showed the beneficial effects of HIP on the density, microstructure and thus the mechanical properties. For example, [38] reported that the use of HIP allowed for an increase in the printed tungsten part density, a reduction in voids and cracks, improvements of the microstructure (more equiaxed structure, larger grain size) and an increase in the thermal conductivity. Furthermore, ref. [44] also reported an increase in the UCS and a decrease in microhardness after the HIP treatments.

Finally, B. Vrancken et al. [37] highlighted that cracks occur at temperatures of the same order of magnitude as the tungsten DBTT (around 570 K), regardless of the laser energy density. To reduce the DBTT and therefore improve the LPBF manufacturability of tungsten parts, adding rhenium could be suggested [64,65,66]. This approach was implemented in [67] for three different alloy compositions (W-10%Re, W-3%Re and W-1%Re) and compared to pure tungsten. This study particularly highlights a reduction in the size of cracks with an increase in the rhenium percentage. In addition to this advantage, it has been shown that combining tungsten with rhenium can also improve the material hardness and strength [66,68,69]. It was also reported that the beneficial effect of rhenium addition occurs when its concentration is either low (5 at%) or close to the maximum solubility of rhenium in tungsten (25 at%) [66].

## 5. Conclusions

- A numerical model based on simple analytical considerations of the LPBF process was developed to calculate the melt pool dimensions and predict the part density. The melt pool calculations have been validated using experimental data taken from the literature, and the density predictions were validated through experiments.
- Structural analyses revealed that the specimens are crack-free, but some of them possess unfused powder particles inside the pores, which means that the Archimedes’ measurements overestimate the part density.
- A strong link between the physical and mechanical properties of the printed specimens and the process parameters was established, namely that:
  a. Density and strength first increase and then decrease according to the h/t ratio, whatever the selected VED*-*BR set.
  b. A ratio of h/t = 2.7 was established as the best for all the VED-BR sets.
  c. A VED=417 J.mm^−3^ − BR=1.62 cm^3^.h^−1^ parameters set was found to lead to the best properties irrespective of the h/t value.

Thus, the best properties (density = 93.2%, UCS = 867 MPa and δ = 6.9%) were obtained for P = 188 W, v = 188 mm/s, h = 80 µm and t = 30 µm.

The above trends were also predicted by the model, meaning that the latter is relevant to predict the printed density as a function of the process parameters, at least in the framework of this study. If a more extensive validation of the model is carried out (different printers and powder feedstocks), this model could be generalized for use with other materials and other LPBF printers.

- Using the defined optimal set of parameters, gyroid lattice structures and wall and gap artifacts were printed. The comparisons between the nominal and the measured characteristics enable the conclusion that this set of parameters also leads to good printability and small printable limits.
- Comparisons with the literature followed by numerical studies using the developed model revealed that the printed density can be improved when considering thinner layers, more powerful LPBF devices, post-treatments and alloying. Also, the processing parameters must be specifically optimized for each individual printer and powder feedstock when such changes are made.

## 6. Future Work

The results of this study offer several avenues for future development. First, the numerical model needs to be tested and validated on different printers, powder feedstocks and materials in order to attest its robustness. Once this validation completed, it could become a powerful tool for the implementation of new LPBF-ready materials. Moreover, post-treatment and/or alloying studies should be carried out to improve the mechanical properties of tungsten-based parts produced by LPBF. Other mechanical testing routines (elevated temperature testing, fatigue, etc.) could also be envisaged to complete the mechanical characterization of printed tungsten parts. Finally, other geometries could be printed to enrich the printability study of this material.

## Figures and Tables

**Table 1:** Rheological properties of the −41 + 20 µm tungsten powder (indexes adopted from [30]): BD (bulk density), CI (compressibility Index), PD (pressure drop), SE (specific energy), AE_10_ (aeration energy at 10 mm.s^−1^ air velocity), BFE (basic flow energy), CC (Cohesion coefficient).

		FT4 Rheometer						
Carr Index	Hausner Ratio	BD (g.cm^−3^)	CI (%) at 15 kPa	PD (mBar) at 15 kPa	SE (mJ.g^−1^)	AE_10_ (mJ)	BFE (mJ)	CC (kPa)
0.00	1.00	12.1 (±0.375%)	1.82 (±5.08%)	14.6 (±3.88%)	1.62 (±0.523%)	23.0 (±1.7%)	1182 (±2.09%)	0.38

**Table 2:** Property variation ranges considered as model inputs; material density is taken from [1], and bulk density is extracted from the rheometer test (BD, Table 1); calculated properties are obtained using equations (in brackets); remaining properties are taken from [36].

Temperature of Application (K)	Material Density (kg.m^−3^)	Bulk Density (kg.m^−3^)	Specific Heat Capacity (J.kg^−1^.K^−1^)	Thermal Conductivity (W.m^−1^.K^−1^)	Electrical Resistivity (nΩ.m)	Absorptivity (%)
293 (RT)–3683 (TM)	19,254	12,100	131–296	47–74 Equation (9)	53–1219	18–51 Equations (6)–(8)

**Table 3:** Coefficients of the polynomial function linking dimensionless melt pool metrics to printed density.

$a_{00}=16.8$	$a_{10}=94.6$	$a_{01}=40.1$	$a_{20}=-53.6$	$a_{11}=-7.5$
$a_{02}=-24.0$	$a_{30}=14.4$	$a_{12}=3.7$	$a_{03}=6.0$	$a_{40}=-1.5$
$a_{31}=0.5$	$a_{22}=-0.8$	$a_{04}=-0.6$		

**Table 4:** DOE: Parameter sets definition.

	A	B	C	D	E
*VED*, J.mm^−3^	1170	750	750	417	417
*BR*, cm^3^.h^−1^	0.43	0.43	0.86	0.86	1.62

**Table 5:** Optimized sets of printing parameters and corresponding printed densities for 200 and 400 W LPBF printers (numerical predictions).

Layer Thickness (µm)	Device	Power (W)	Speed (mm.s^−1^)	Hatching Space (µm)	VED-BR (J.mm^−3^; cm^3^.h^−1^)	Predicted Density (%)
20	200 W	188	375	60	(417; 1.62)	96.9
	400 W	400	750	60	(444; 3.24)	98.7
30	200 W	188	200	75	(417; 1.62)	93.7
	400 W	400	333	90	(444; 3.24)	98.6

## Data Availability

Data are contained within the article.

## Appendix A

**Table A1:** Printed specimens: nomenclature, plan of experiments, predicted density, measured density and error between the densities.

Name	Hatching Space (µm)	Power (W)	Speed (mm.s^−1^)	Volumetric Energy Density (J.mm^−3^)	Built Rate (cm^3^.h^−1^)	Predicted Density (%)	Image Analysis Density (%)	Absolute Error between Densities (%)
A-20	20	140	200	1167	0.43	86.9	87.4	0.5
B-20		89	197	750	0.43	72.8	82.6	9.9
C-20		179	398	750	0.86	89.7	84.7	5.0
D-20		100	398	417	0.86	72.8	69.5	3.3
E-20		188	750	417	1.62	87.2	78.3	8.9
A-30	30	140	133	1167	0.43	90.2	87.7	2.5
B-30		89	131	750	0.43	78.2	75.9	2.3
C-30		179	265	750	0.86	93.8	92.4	1.4
D-30		188	500	417	0.86	78.2	82.1	3.9
E-30		100	265	417	1.62	92.2	82.6	9.6
A-40	40	140	100	1167	0.43	91.9	88.3	3.6
B-40		90	100	750	0.43	77.4	78.5	1.2
C-40		179	199	750	0.86	94.8	89.8	5.0
D-40		188	375	417	0.86	77.4	80.5	3.1
E-40		100	200	417	1.62	93.4	91.4	1.9
A-60	60	140	67	1167	0.43	91.1	90.2	0.9
B-60		90	67	750	0.43	74.6	90.2	15.6
C-60		179	133	750	0.86	95.1	88.2	6.9
D-60		100	133	417	0.86	78.3	78.4	0.1
E-60		188	250	417	1.62	94.2	92.1	2.1
A-80	80	140	50	1167	0.43	91.2	91.2	0.0
B-80		180	100	750	0.43	72.1	84.3	12.3
C-80		89	49	750	0.86	94.2	89.2	5.0
D-80		188	188	417	0.86	78.2	85.9	7.7
E-80		100	100	417	1.62	94.2	93.2	1.1
A-100	100	140	40	1167	0.43	89.7	91.2	1.5
B-100		90	40	750	0.43	70.0	83.5	13.5
C-100		179	80	750	0.86	93.9	93.5	0.4
D-100		188	150	417	0.86	76.3	81.5	5.2
E-100		100	80	417	1.62	93.1	91.8	1.3

## Appendix B

**Table A2:** Printed specimens: nomenclature, pore density, pore size, yield strength at 0.2% offset, ultimate compression strength, strain under maximum load and maximum compression strain.

Name	Pore Density (p.mm^−2^)	Pore Size DE50_p_ (µm)	YS at 0.2% (MPa)	UCS (MPa)	Strain under Maximum Load (%)	Maximum Compression Strain (mm.mm^−1^)
A-20	101	95	357	460	2.9	0.10
B-20	166	85	222	335	3.8	0.08
C-20	123	120	315	415	2.6	0.07
D-20	128	120	274	392	4.2	0.11
E-20	92	125	320	425	3.3	0.09
A-30	195	80	418	592	5.4	0.13
B-30	120	100	258	449	6.0	0.11
C-30	157	95	537	705	2.9	0.07
D-30	119	85	288	459	5.7	0.11
E-30	75	120	376	542	4.7	0.10
A-40	96	85	323	549	5.3	0.12
B-40	187	85	275	492	6.8	0.12
C-40	114	90	547	635	3.5	0.11
D-40	292	80	376	631	8.1	0.14
E-40	117	95	544	694	3.5	0.09
A-60	112	90	513	739	6.7	0.13
B-60	209	75	396	629	7.7	0.14
C-60	116	90	526	652	3.8	0.09
D-60	225	80	394	599	7.6	0.13
E-60	106	90	669	810	3.3	0.09
A-80	116	70	458	735	8.4	0.14
B-80	185	75	347	617	10.2	0.16
C-80	149	85	540	651	2.9	0.08
D-80	143	70	396	671	11.0	0.18
E-80	76	80	676	867	6.9	0.15
A-100	192	60	521	799	9.6	0.16
B-100	244	70	368	655	10.7	0.17
C-100	195	65	584	803	6.6	0.12
D-100	206	85	449	662	9.0	0.17
E-100	158	75	591	777	5.9	0.11
